# Sext Dissemination: Differences across Nations in Motivations and Associations

**DOI:** 10.3390/ijerph18052429

**Published:** 2021-03-02

**Authors:** Elizabeth M. Clancy, Bianca Klettke, Angela M. Crossman, David J. Hallford, Dominika Howard, John W. Toumbourou

**Affiliations:** 1 School of Psychology, Deakin University, 221 Burwood Hwy, Burwood East 3125, Australia; bianca.klettke@deakin.edu.au (B.K.); david.hallford@deakin.edu.au (D.J.H.); dominika.howard@deakin.edu.au (D.H.); 2 Department of Psychology, John Jay College, City University of New York, 524 West 59th Street, New York, NY 10019, USA; acrossman@jjay.cuny.edu; 3 School of Psychology, Deakin University, 1 Gheringhap Street, Geelong 3220, Australia; john.toumbourou@deakin.edu.au

**Keywords:** sexting, sext dissemination, young adult, technology-facilitated sexual abuse, cross-national

## Abstract

Sext dissemination presents policy and legislative challenges given its potential psychological, social, and legal harms. We report on a cross-national comparison of sext-image dissemination in a large sample of 1148 young adults aged 18–29 years (M = 22.54, SD = 2.50, 53.0% women, 47.0% men), either U.S. (53.8%) or Australian (46.2%) residents. The results indicate that 14% of young adults disseminated sexts, with no difference by gender or country. Over 50% of respondents indicated that the last time they received a disseminated sext, it was unexpected or unwelcome, with women twice as likely as men to receive unwelcome sexts. The most frequent motivations for sext dissemination were similar cross-nationally, relating to the attractiveness of the person depicted, as a joke, to gossip, because it was not a big deal, bragging, roasting or teasing, and to increase social status. Motivations of attractiveness, bragging, or social status were more commonly endorsed by men, while women endorsed reasons around gossip or roasting/teasing. Unique predictors of sext dissemination included U.S. residence, requesting sexts, receiving disseminated sexts, having one’s own images disseminated, and more positive subjective norms to dissemination, and there was a country–gender interaction, where Australian women and U.S. men were more likely to disseminate sexts than then U.S. women or Australian men. The findings have implications for prevention programs seeking to address harmful online sexual interactions, including addressing respect, consent, and subjective norms supporting non-consensual dissemination.

## 1. Introduction

It has been a decade since the portmanteau term “sexting” was accepted into the Concise Oxford Dictionary [1]. Since then, this topic has continued to gather copious attention from the media and researchers, particularly in relation to the potential harms that may occur due to sexting. Defined as the sending, receiving, or forwarding of sexually explicit messages, images, or photos to others through electronic means [2], sexting is an increasingly normative sexual behaviour in emerging adulthood [3], and part of interpersonal sexual communication [4]. A recent meta-analysis [3] estimated that 38% of young adults have sent sexts, 42% have received sexts, 48% have engaged in reciprocal sending, and 15% have forwarded sexts without consent. These rates have been increasing in more recent years, with some studies finding that 70% of young adults report sending sexts, and in excess of 80% receive sexts [5,6].

While much research has investigated potential harms associated with sexting behaviours, particularly those associated with the sending and receiving of sexts, less focus has been paid to the behaviour of sext dissemination. Sext dissemination is defined as the distribution of sexts to people beyond the original intended audience. The limited research conducted in this area to date has largely focussed on prevalence rates and potential consequences for those whose images are distributed non-consensually—that is, without their knowledge and/or consent [7]. These consequences include negative impacts on trust, increased anxiety and depression, post-traumatic stress disorder, and suicidal thoughts [8]. Given the potential harms associated with non-consensual sext dissemination, understanding those who engage in this behaviour is critical. Research regarding the decision-making processes, motivations, and risk and protective factors of perpetrators of sext dissemination is vital. This would help to identify causal factors in sext dissemination, and inform the development of evidence-based prevention initiatives.

### 1.1. Implications for Perpetrators

As noted above, harms associated with non-consensual sext dissemination are often considerable for victims. However, consequences for perpetrators can also be severe, with many Western jurisdictions legislating legal or criminal sanctions for dissemination. For example, in several U.S. states [9] and multiple jurisdictions across Australia [10], image-based non-consensual sext dissemination by adults has been criminalised. Moreover, in one Australian state (Victoria), even threats to disseminate an image-based sext are punishable by imprisonment [11] (S.41DB). Sext dissemination may incur additional penalties under child pornography laws if distributed images depict minors or are sent to minors, regardless of the age of perpetrators [7].

For adults, engagement in sext dissemination may also have broader negative implications. For example, the public careers of U.S. Republicans Nick Sauer [12] and Eric Greitens [13], and an Australian political staffer [14], all ended after accusations of engaging in non-consensual sext dissemination, specifically revenge pornography (the dissemination of images which are obtained consensually within a relationship, but posted or published more broadly in the context of an acrimonious relationship breakdown [7]). Civil litigation has also been launched in relation to military [15] and law enforcement [16] figures. Those who disseminate sexts may also receive negative feedback from peers who disapprove of such behaviours [17].

### 1.2. Motivations for Sext Dissemination

To understand sext dissemination behaviours, it is important to consider the underlying motivations. Only four empirical studies to date have explicitly explored sext dissemination motivations: three with young adult populations [5,6,18] and one investigating adolescents [19]. Among young adults, dissemination prevalence rates were similar across genders, ranging from 16 to 19%. For adolescents, 15% reported having shared images, with boys significantly more likely (21%) than girls (9%) to have done so [19]. Motivations or rationalisations for sext dissemination were generally self-reported as relatively innocuous, with the most frequent self-ascribed motivations including “for fun, as a joke”, that it “was not a big deal”, or “because the person was hot” [5,6,18]. Similar motivations were reported by adolescents, specifically for fun or as a joke, showing off, and not realising what they were doing [19]. It can be argued that some of these reasons, especially “not a big deal” or “as a joke” are less behavioural motivations, and more a subjective judgement about potential impacts of dissemination. However, those engaged in dissemination seem to perceive such behaviour as socially desirable and/or amusing, or at least relatively harmless. Considering gender differences, whilst prevalence rates are similar (less so among adolescents), motivations appear to vary, with men more likely to endorse social status motivations and women more likely to endorse humour [5,6]. Qualitative data [6] also indicate that a small but significant group, almost exclusively women, report having received unwanted or unwelcome images which they then forwarded to friends or peers as proof of harassment. Interestingly, recent research [20] investigating responses to unsolicited explicit material shared via social media indicated that women find unsolicited sexual content less funny or exciting and more disturbing, than men. There was also some degree of desensitisation, with more frequent recipients finding such content funnier than those with minimal or no exposure.

Decisions to disseminate sexts have been explained with reference to the theory of reasoned action [21], which argues that individual intentions to engage in specific behaviours are based on an evaluation of relevant attitudes and subjective norms. Specifically, positive attitudes in relation to the outcomes of disseminating and subjective norms that dissemination is normalised and accepted by peers are both related to an increased likelihood of disseminating [5]. These norms may be formed and reinforced through having received disseminated sexts and having had one’s own images shared with others [6].

### 1.3. Cross-Cultural Studies of Sext Dissemination

To understand whether sexting is influenced by international versus national trends, it is valuable to conduct cross-nation studies. Previous studies investigating motivations for sext dissemination have been limited to single nation samples (from the United Kingdom, Australia, and Switzerland). No studies have directly compared countries in terms of sex dissemination motivations and related factors. In fact, cross-cultural investigations of sexting behaviours in general are limited. To the authors’ knowledge, only two major cross-national published studies of sexting behaviour have investigated the sharing of images, or dissemination. Only one of these studies included adult participants, and none of the studies investigated sext dissemination motivations. In a study of sexting within adolescent romantic relationships across five European nations, researchers [22] found that between 13 and 24% of adolescents reported having shared images with others, whilst 8–32% of adolescents reported that their partners had shared images they had sent, with proportions varying by nation and no aggregate levels reported. More recently, [23], a study of adolescents and young adults across 10 nations over four continents, reported rates of non-consensual sext dissemination, including privately sending and publicly posting sexts of someone without their consent. Considerable variation was noted across countries, with reported rates of ever having engaged in non-consensual dissemination ranging from 9% in Turkey to 24% in Uganda.

These cross-national differences in reported rates bring into question underlying assumptions of homogeneity regarding both the prevalence and motivations for sext dissemination. In particular, such differences suggest that the same risk and protective factors may not apply across countries, thus limiting the generalisability of prevention and/or intervention campaigns. For example, it is often assumed that Western norms and attitudes towards sexting behaviours are relatively homogenous across Europe, North America, and Australasia, or that U.S.-based research regarding sexting is applicable to other Western contexts, such as Australia. However, such assumptions fail to take into consideration the potential for important differences in contextual factors.

One key difference which may impact sexting behaviours, and particularly sext dissemination, relates to the content of school-based sexual health and relationships education, specifically around sexting and online interactions. Comparison between the U.S. and Australia offers some striking differences. Australian jurisdictions typically mandate comprehensive sexuality and relationship education in school curricula. This education covers not only topics such as sexually transmitted disease and safe sex practices, reproduction, and birth control, but also social issues around managing peer influence, relationships and consent, and decision making [24]. More recent educational materials have an increased focus on online as well as offline behaviours [25]. In contrast, U.S. educational requirements vary widely across states, with several states emphasising abstinence as the preferred approach to preventing sexually transmitted infections, and have limited information about safe sex practices [26,27], whilst broader discussion of sexual practices in educational contexts remains controversial in some states [28].

The initial U.S. National Sexuality Education Standards (NSES), which provided guidance on essential minimum, core content and skills for K-12 age groups [29], did not include the words “online”, “internet”, or “sext”, and only mentioned “consent” six times. Recent updates [30] still make no mention of “internet”, refer to “online” once, and includes “sext” only 6 times, solely as legislative references. In contrast, Australia’s Respectful Relationships curriculum material [31] provides explicit teaching materials with multiple sexting scenarios and negotiation of online consent. Whilst comparative evaluations of these programs are unavailable, explicit reference to online behaviours and sexting is limited in the U.S. mandated curriculum. In contrast, Australian students discuss and consider scenarios such as being asked or pressured for explicit images, having explicit photos taken non-consensually, sending nudes, and having those images shared with others consensually or non-consensually.

Other evidence points to differences in offline sexual behaviours between Australian and U.S. cohorts [32]. In a cross-national longitudinal study, U.S. adolescent girls were more likely to be sexually active in early adolescence (12–13 years) than their Australian peers. However, by mid adolescence (~15 years), Australian girls reported being sexually active and having more partners than their U.S. counterparts. Given differences in education programs and offline sexual behavioural differences, investigation into the rates and motivations for engaging in online sexual behaviours may therefore also show cross-national differences and indicate different approaches to prevention.

Another construct which may explain differences in sext dissemination behaviours is consent. Those who share images with consent from those depicted should be considered as engaging in a very different behaviour from those who disseminate images non-consensually, which would potentially constitute image-based sexual abuse. Some of the observed differences in consensual and non-consensual dissemination behaviours could be explained by unconditional respect for others. Unconditional respect [33] can be considered a fundamental attribute, whereby respect is owed to all others, regardless of their status, achievements, or activities, and cannot be earned or taken away. This conceptualisation of respect for the autonomy and integrity of others is anticipated to result in decreased rates of dissemination, if dissemination is understood as harmful for the person depicted. As such, the concept of unconditional respect may contribute to explanations of individual differences in sext dissemination. In addition, further exploration of cross-national differences in respect may reveal that they are consistent with differences in curriculum emphasis and help to explain differences in observed behaviours between the nations.

### 1.4. The Current Study

In summary, prior empirical investigations of sext dissemination have assessed its association with personality variables, subjective norms and attitudes, and behavioural and motivational factors. Whilst the literature is limited, there appear to be relationships between dissemination and behavioural norms, such as receiving disseminated images and normalising dissemination, fostering the perception that it is not a big deal [5,6,18]. Despite evidence that dissemination rates differ across countries, no cross-national comparisons of dissemination motivations are available.

The current study aimed to explore whether, in addition to previously observed gender-based motivational differences, there are cross-national differences between U.S. and Australian young adults in rates and motivations for sext dissemination. Secondly, we aimed to determine whether any such differences could be attributed to modifiable factors including behavioural associations, subjective norms and attitudes towards dissemination, and unconditional respect for others. Based on prior findings, we hypothesised that rates of dissemination would be similar across genders, but that motivations would differ between men and women. We also hypothesised that U.S. participants would be more likely to engage in sext dissemination, based on reduced explicit online sexual education content.

## 2. Materials and Methods

### 2.1. Participants

Participants in this study included 1148 young adults aged 18 to 29 years (M = 22.54, SD = 2.50, 53.0% women, 47.0% men). Participants were residents of either the U.S (53.8%) or Australia (46.2%). There was no significant difference between the samples on gender (χ^2^ (1) = 3.739, *p* = 0.053), although the U.S. sample was older (M = 23.37 yrs., SD = 2.09) than the Australian sample (M = 21.57 yrs., SD = 2.59) overall, t (1146) = −13.01, *p* < 0.001. Most respondents (80.7%) reported their sexual orientation as heterosexual, while 14.5% were bisexual, 3.7% were lesbian or gay, and 1.0 % were unwilling to disclose. Participants were not asked to specify their relationship status. However, the vast majority (81.8%) reported being currently sexually active, with 17.19 years (SD = 2.44) being the average age of becoming sexually active.

### 2.2. Materials

Sext dissemination behaviours questionnaire: this study adapted a previous sext dissemination questionnaire to focus on specific aspects of sexting [6]. For the purposes of this study, all sexting-related questions focused on images, referring to sexts as “sexually explicit images, sent, received, or shared via mobile phone messaging or apps”. Sexting behaviours were assessed via questions about engagement in requesting and receiving sexts, sending images of themselves, and dissemination. Specifically, participants were asked about both disseminating and receiving disseminated sexts; “Has someone ever forwarded you an image-based sext via text or mobile app that was not originally intended for you?” (Yes/No) and “Have you ever received an image-based sext intended for yourself which you subsequently showed/sent to another person?” (Yes/No). Participants who responded yes were then asked if receipt of these images was unwanted and/or unwelcome, how often they had disseminated images, and to estimate to how many people they had sent the sext.

Participants were also asked about motivations for sharing sexts: “What were the reasons why you decided to share the sext message with others?” Based on prior work [5], the following options were provided; as a joke, to be funny: to get attention/praise; because another person asked you to; to improve your social status amongst peers; because you felt pressured to do so; to get the recipient into trouble; to get back at the person; i did not think it was a big deal; because the person in the image was hot; out of spite; to gossip; to initiate sexual contact; to roast or tease the person depicted; to get revenge; to brag. Participants also had the option of “other” and could provide open-ended responses.

Finally, participants were also asked whether they knew of their own images having been shared with others; “Have you ever sent an image-based sext of yourself that was subsequently forwarded (to your knowledge?)”. If they were aware of this occurring, they were also asked whether they had consented; “Had you given permission for this image to be forwarded?” with responses options of Yes/No. For a full version of the questionnaire, please see Supplementary Materials.

Subjective norms and attitudes: to assess attitudes and subjective norms concerning sext dissemination behaviours, a 5-item scale was adapted from prior investigations of sexting [34]. The initial 7-item scale referred to sexting behaviours, so items were adapted to reflect sext dissemination, based on prior item wording [5]. Two items (Items 3 and 4 from the initial adaptation) were poorly correlated with the overall scale, and were removed for this analysis (refer to Appendix A, Table A1, for details). A sample item is “Sharing sexually explicit images via text or mobile app of others can enhance social status”. Participants indicated their agreement with statements on a 5-point Likert scale from 1 (strongly disagree) to 5 (strongly agree). After reverse coding relevant items, responses were summed together, with higher scores indicating generally positive subjective norms and attitudes towards sext dissemination. Internal reliability was acceptable, with a Cronbach’s alpha of 0.67.

Respect for others was assessed using 10 items drawn from the Unconditional Respect for Persons scale (URfP) [33]; A sample item is “Treating all people with respect is a vital part of our relationships with others”, and responses were scored on a Likert scale ranging from 1 (Not at all true) to 7 (Very true). Two items relating to physical punishment for crimes were removed from the initial 12-item scale (Q2, Q7). Once relevant remaining items were reverse coded, a mean score was calculated overall, with higher scores indicating greater levels of unconditional respect. Internal reliability in this sample was good, with a Cronbach’s alpha of 0.86 exceeding published scores from the initial validation study of 0.75 [33].

### 2.3. Procedure

After obtaining ethics approval from Deakin University’s Human Research Ethics Committee, participants were recruited through social media (Facebook, Instagram, and Reddit, *N* = 310). To achieve balanced samples by gender and country for analyses, additional participants were recruited from survey aggregator sites Amazon MTurk (*N* = 702) and Prolific (*N* = 136) for both Australian and U.S. cohorts. In planned contrasts between social media and survey aggregator participants, those recruited via social media were more likely to be women (t (1145) = 20.26, *p* < 0.001), and resident in Australia (t = −34.51, *p* < 0.001), whilst there was no significant difference in age (t = −1.93, *p* = 0.06). Social media participants were also more likely to have disseminated sexts than those from survey aggregator sites (t = 2.24, *p* = 0.026), although the absolute value of this difference was small (Facebook 15%, Survey aggregators 13%).

Advertisements informed participants that the study aimed to explore factors that can influence sexting behaviours, was open to adults aged 18–29 years, regardless of whether they had sexted or not, and emphasised response anonymity. Potential participants were directed to an online survey collector, where they could review a brief study description and plain language statement. The plain language statement provided example questions of the survey and included information about the study itself and support information in case of any potential distress. The statement also confirmed that participants could withdraw at any point prior to completing the survey, but that once responses were submitted, they could not be withdrawn, as all responses were completely anonymous. Participants were able to review this information and record their consent electronically, prior to commencing the survey. The survey took approximately 15–20 min to complete, with no incentive offered for general social media participants. MTurk participants received a payment of 1USD per survey completion, whilst Prolific users received a payment of 1GBP for completion. Survey responses were gathered between July and November 2019.

### 2.4. Analyses

Data cleaning involved removing incomplete or non-random invalid responses. Following this, outliers were trimmed via Winsorization to the 5th and 95th percentile [35]. Descriptive statistical analyses were used to review sample and variable characteristics. Analysis of difference by gender was conducted via chi-squared analyses where cell counts exceeded five, and bivariate correlations between independent variables and sext dissemination. Binomial multivariate logistical regressions were used to probe the association of behavioural and personality variables with sext distribution. Only variables with a significant bivariate relationship with disseminating sexts were included in regression analyses.

## 3. Results

### 3.1. Descriptive Statistics

Table 1 provides descriptive statistics overall, by country and by gender, whilst Table 2 presents the same data by gender within each nation for key study variables. The vast majority of the sample (86.4%) reported having received sexts, with no difference by country. More than half had received unwanted or unwelcome sexts, with women 2.5 times as likely as men to receive unwanted or unwelcome sexts, but with no difference by country. Almost two-thirds of respondents had requested a sext. U.S. respondents were significantly more likely to have requested a sext than Australian respondents. Additionally, men across both countries were significantly more likely to have requested a sext than women. More than two in three participants had sent sexts of themselves, with women significantly more likely to have sent a sext.

Regarding sext dissemination behaviours, 14.1% of participants had disseminated sexts to others, on average close to four times, and sent to more than two people on average, with no differences across gender or country. U.S. respondents tended to share disseminated sexts with more people than Australians, but this difference was not significant. Almost one third of participants had received disseminated sexts, again with no difference by gender, but Australians were 40% more likely to have received disseminated sexts than U.S. respondents. Both men and women reported that the images they disseminated were generally received from someone of a different gender, with women significantly more likely to report this than men. Disseminated images were typically forwarded to those of the same gender. However, women were more than twice as likely as men to report that the sexts they disseminated to others were unexpected or unwelcome. In addition, women reported being twice as likely to receive disseminated images that were unwanted or unwelcome. One in eight respondents knew of their own sexts being disseminated, with men (29.4%) being more than four times as likely as women (6.9%) to give consent for this, and U.S. respondents being three times as likely (23.2%) to provide permission as Australians (7.2%).

No published norms are available for measures of unconditional respect or subjective norms and attitudes. However, scores on the Unconditional Respect for Persons scale were indicative of positive levels of unconditional respect, with mean scores greater than a neutral score of 4. Women reported higher levels of unconditional respect than men overall, whilst Australian respondents reported higher levels of unconditional respect than U.S. respondents. Considering subjective norms and attitudes (SNA) towards sext dissemination, mean responses indicated a mild rejection of positive attitudes towards dissemination. There was no significant difference in SNA overall by country, but men had significantly less negative attitudes to dissemination overall and in both Australian and U.S. cohorts separately.

### 3.2. Correlational Analyses

Correlations between key variables of interest for each country are presented in Table 3. Sext dissemination was not associated with age or gender for either cohort, but was positively associated with most general sexting behaviours, other dissemination experiences, such as having received disseminated images and having one’s own images shared with others, and positive subjective norms and attitudes to dissemination. There was a negative relationship between dissemination and having received unwanted or unwelcome disseminated images. Comparing the two cohorts, for U.S. respondents, dissemination was associated with having consented to have one’s own images shared with others, and with lower levels of unconditional respect, whilst there was no such association for Australian respondents. In contrast, for Australian participants, there was a negative correlation between dissemination and having received disseminated images that were unwanted or unwelcome.

### 3.3. Dissemination Motivations

Motivations for sext dissemination were analysed by exploring gender differences overall, and for each nation separately (Table 4). The most frequent motivations for dissemination were that the person depicted was hot, as a joke, to gossip, because it was not a big deal, to brag, to roast or tease the individual and to increase social status. Men were more likely to endorse dissemination because the person was attractive, or for bragging or social status, whilst women were more likely to endorse gossip as a motivation. When compared by gender separately for each country, both Australian and U.S. men were significantly more likely than women to endorse reasons of the person being hot, whilst U.S. women were more likely to endorse motivations related to gossip. Other differences were noted, but cell counts were too small to report statistical differences.

### 3.4. Regression

A hierarchical logistic regression was used to determine the unique and shared contributions of factors significantly correlated with sext dissemination overall, whilst controlling for age, gender, and country. Some variables were not included in this regression (receipt of unwanted images, having disseminated unwanted images, and consent for own image being disseminated) as inclusion would substantially reduce sample size and power. Due to observed differences in subjective norms and dissemination patterns by gender, interactions between gender and country and gender and subjective norms were also included in the hierarchical regression.

The overall model was significant: χ (11) = 127.56, *p* < 0.001, and explained 28% of variance (R^2^Nagelkerke = 0.277). As shown in Table 5, unique significant predictors of sext dissemination were country (U.S. participants more likely than Australians), having requested sexts, having received disseminated sexts, having had one’s own images disseminated, and more positive subjective norms and attitudes towards dissemination. The interaction between gender and country was significant (χ (1) = 8.40, *p* = 0.004), with Australian women being more likely than Australian men to disseminate, whilst U.S. men were more likely than U.S. women. However, this only explained an additional 2% of variance in the model. The interaction between gender and more positive subjective norms and attitudes towards dissemination did not significantly improve the model (χ (1) = 0.30, *p* = 0.585). Interactions between gender and more positive subjective norms and attitudes towards dissemination (χ (1) = 0.30, *p* = 0.585), and between country and more positive subjective norms and attitudes towards dissemination (χ (1) = 1.12, *p* = 0.291) were not significant, and did not improve the model overall.

## 4. Discussion

The primary aim of this study was to explore potential cross-national differences in relation to sext dissemination, in addition to replicating findings of gender-based motivational difference. The secondary aim was to determine whether any such differences could be attributed to modifiable factors including behavioural associations, subjective norms and attitudes towards dissemination, and unconditional respect for others. Our hypotheses, that dissemination rates would be similar across genders, but motivations would differ, whilst overall U.S. participants would be more likely to engage in dissemination than Australian participants, were partially supported.

Overall, approximately one in seven respondents reported having disseminated sexts, with no differences by country or gender. This is consistent with previous studies that found similar rates of dissemination behaviours, but no difference by gender [5,6,18,19], but does not support our hypotheses regarding cross-national differences. Respondents of both genders who had disseminated sexts reported that they typically received the image from someone of a different gender, but generally forwarded to those of the same gender. However, women were almost twice as likely as men (67% vs. 35%) to report that the sexts they disseminated to others had been unexpected or unwelcome when they had received them. Given that this sample was predominantly heterosexual, and images were largely of someone of a different gender, it appears that sexual orientation is not the basis for women being more likely to report that they were disseminating unwelcome or unwanted sexts.

Almost twice as many respondents (30% overall) reported having received disseminated sexts from others, which is consistent with reports that those who disseminate send to multiple recipients. There was no difference in receiving disseminated sexts by gender, but Australian respondents were around 40% more likely to have received a disseminated sext than U.S. respondents. Notably, more than half of all those receiving disseminated sexts reported that the last time they received a disseminated sext, it was unwanted or unwelcome, with these rates significantly higher for U.S. than Australian respondents.

It is important to note that both receiving and forwarding unwanted or unwelcome disseminated sexts were significantly different by gender, with women twice as likely to report receiving unwanted disseminated sexts, and up to seven times more likely (for Australian women versus men) to report having disseminated images that were unwanted or unwelcome. These findings are consistent with prior qualitative findings [6] that women report disseminating images which have been received unexpectedly, potentially as evidence of harassment. They are also consistent with recent research [20] that suggests that women find unsolicited content to be less funny or exciting, and more disturbing, than men. As such, dissemination of this unwanted material may relate to mitigating distress or seeking reassurance from peers, and this experience is more common for women.

Similar to prior findings [6], one in eight participants overall reported being aware that their sexts had been disseminated to others, with women significantly more likely to report this than men. It is likely that such responses underestimate the prevalence of dissemination victimisation, as many respondents may be unaware of their intimate images being shared with others. Importantly, only 15% of individuals aware of having had their images shared had provided consent for this, with men four times more likely to provide such permission than women. Consent was also significantly more common for U.S. respondents (23% giving permission) than Australian respondents (7%) overall, although the reasons for this are unclear. These findings speak to a large proportion of sext dissemination, although not all, being non-consensual, and highlight the importance of determining whether dissemination is consensual or not in future research.

With regard to subjective norms and attitudes towards dissemination, there was no difference across countries, but men had significantly more positive attitudes towards dissemination than women. This may reflect peer groups norms, whereby men are less exposed to the potential for harms that may be experienced. Specifically, sexual double standards have resulted in women being more harshly criticised when images they send are shared more broadly [36,37].

Given that the majority of sexts were disseminated without prior consent, it is critical to consider the motivations of those who share such images. Overall, self-attributed motivations differed more by gender than country. Seeing the person depicted as attractive or “hot” was the most common motivation cited, and was higher for men than women in both samples. The second most common reason overall, and highest for women, was “as a joke”, and there was no gender difference in either cohort. All groups endorsed seeing dissemination as “not a big deal” relatively frequently, which is consistent with other findings [5,6,18,19] that those who engage in dissemination rarely perceive this behaviour as harmful. However, as noted earlier, seeing dissemination as “not a big deal” or “as a joke” may be less reflective of reasons for engaging in a behaviour or motivations, and more correctly indicate a subjective judgement about the low potential impact of dissemination. Those who see dissemination in this light may engage in little reflection or pause prior to disseminating images.

Explicit motivations of spite or revenge were relatively infrequent for both cohorts and genders, consistent with prior studies [5,6], and suggesting that narratives that equate non-consensual dissemination with so-called “revenge pornography” fail to encompass the breadth of this behaviour. However, adding to prior findings, women in particular were more likely to report motivations related to gossiping or roasting/teasing others. This may be an extension or more specific example of seeing such behaviour as “a joke”, or may reflect a somewhat less innocuous motivation, although not to the extent of revenge. It may also indicate that women, relative to men, use the distribution of sexual images of men (and women) as a way of demoting or insulting the social status of others, while also minimising or downplaying the intention and consequences of their own actions. The overall order of motivations was similar in both countries, suggesting that similar motivations operate within both contexts.

Lastly, a regression analysis explored the relative contributions of predictors of dissemination. Unique predictors of increased likelihood of sext dissemination were being from the U.S., having requested sexts, having received disseminated sexts, or having had one’s own images disseminated and more positive subjective norms and attitudes towards dissemination, as well as an interaction between gender and country, where Australian women were more likely than Australian men to disseminate sexts, whilst U.S. men were more likely to have disseminated sexts than U.S. women. These findings suggest that, overall, dissemination behaviours are associated with normalisation and acceptance of sext dissemination more broadly. Whilst there are some variations between the Australian and U.S. samples, behavioural norms within peer groups appear to be the largest and most relevant predictors of sext dissemination.

Of interest, although levels of unconditional respect were not uniquely associated with dissemination for either cohort or overall, there was a medium-sized negative correlation between respect and subjective norms and attitudes in both countries. In addition, lower levels of unconditional respect were correlated with dissemination for the U.S. sample in particular. These differences may reflect variations in norms that are established and reinforced in adolescence, including through educational programs. Given the age of study participants, it is relevant to examine the curriculum content current at the time of their education. Specifically, the first edition of the U.S. NSES [28] placed minimal emphasis on respect or consent as part of sexual health education programs, especially in comparison to Australian material [31]. Moreover, these are only recommendations and, according to the Centers for Disease Control [38], only 41.3% of school districts reported following the initial standards, with 75% of high schools allowing parents to exclude their children from instruction on human sexuality (80–83% for younger children). Hence, respect for others could promote less favourable subjective norms and attitudes towards sext dissemination, thus serving as a protective factor against non-consensual dissemination, particularly among those who receive explicit education about healthy and consensual sexual behaviours, both online and off.

Whilst this study details findings regarding sext dissemination across two Western nations, particularly normalisation within peer groups, it is important to note limitations. This study was based on a convenience sample, and was not nationally representative for either country. In addition, participants were asked to self-report on their own behaviours. Whilst all efforts were made to ensure the confidentiality of responses, and participants were not identifiable, some may have been reticent to report engaging in behaviours which they deem socially unacceptable. Lastly, this study did not differentiate whether dissemination itself was consensual or non-consensual, which is warranted in the future.

Despite limitations, this study adds to the emerging literature around drivers of dissemination behaviour, and offers the first cross-national study to compare whether determinants of dissemination vary between different Western nations. Based on our results, despite small cross-national differences, with dissemination perpetration being slightly more common in U.S. participants, dissemination receipt being more common in the Australian cohort, and U.S. respondents being significantly more likely to give consent for dissemination of their own images, similarities are more notable. Greater differences can be attributed to gender overall, in relation to experiences of dissemination, the proportion of disseminated images which are unwanted and/or unwilling, and the motivations for such behaviour.

These findings further reinforce prior suggestions [5,6] that the main drivers of dissemination behaviour appear to be behavioural, with normalization via the receipt and exchange of explicit images within peer groups and more positive subjective norms and attitudes towards dissemination being the strongest predictors of engaging in dissemination oneself. That is, having requested sexts from others, disseminated images, having one’s own images shared, and more strongly believing that dissemination has benefits and minimal risks are is associated with engaging in dissemination.

In addition, the role of respect for others may have some value as a protective factor, at least in the U.S. sample. Based on these findings, prevention initiatives, including adolescent relationship education programs, could benefit from increased focus on active consent and respect in relation to online interactions, as part of addressing social norms that such behaviour is subjectively positive for perpetrators, in both U.S. and Australian contexts. This may provide opportunities for more development and evaluation of such programs to determine their impacts in reducing harm.

Future research should continue to investigate drivers of dissemination, given that our model explains only one quarter of the observed variance. Of particular note, unwanted receipt of images was associated with dissemination for a significant proportion of respondents, particularly women, which warrants further investigation via larger samples to determine how such experiences explain specific dissemination scenarios, given prior findings of greater disgust from women [20]. Further exploration of different cohorts, including more cross-national comparisons, experiences of diverse sexual and gender cohorts, and a focus on consensual as opposed to non-consensual dissemination would help to increase understanding. Regardless, sext dissemination occurs with sufficient frequency to warrant inclusion in relationship education programs. Such programs should address the normalization of dissemination behaviours, with an emphasis on active consent and respect, as modern relationships encompass both in person and online communications.

## Figures and Tables

**Table 1:** Sexting behaviours for full sample, and by country and gender overall.

Variable	Full Sample (*N* = 1148)	Aus (*N* = 530)	U.S. (*N* = 618)	Comparison, U.S. to Aus	Men (*n* = 540)	Women (*n* = 608)	Comparison, Men to Women
Received sext	86.4%	84.7%	87.9%	χ^2^ (1) = 2.44, *p* = 0.118	82.6%	89.8%	χ^2^ (1) = 12.33, *p* < 0.001
Received unwanted or unwelcome sext (% of above)	54.8%	56.1%	53.8%	χ^2^ (1) = 0.52, *p* = 0.472	29.3%	75.8%	χ^2^ (1) = 212.95, *p* < 0.001
Requested a sext	50.4%	44.6%	55.2%	χ^2^ (1) = 12.71, *p* < 0.001	59.0%	42.9%	χ^2^ (1) = 29.05, *p* < 0.001
Sent sext (of yourself)	65.2%	64.5%	65.8%	χ^2^ (1) = 0.22, *p* = 0.610	57.4%	72.0%	χ^2^ (1) = 26.24, *p* < 0.001
Received disseminated sext	30.1%	35.8%	25.2%	χ^2^ (1) = 15.24, *p* < 0.001	31.7%	28.8%	χ^2^ (1) = 1.13, *p* = 0.288
Last time received disseminated sext: was receiving image unexpected/unwelcome	52.6%	47.6%	58.6%	χ^2^ (1) = 4.15, *p* = 0.042	37.8%	67.0%	χ^2^ (1) = 29.86, *p* < 0.001
Ever disseminated sext	14.1%	12.8%	15.2%	χ^2^ (1) = 1.33, *p* = 0.248	14.8%	13.5%	χ^2^ (1) = 0.42, *p* = 0.519
Last time disseminated a sext, was receiving image unexpected or unwelcome (% of above)	45.7%	41.2%	48.9%	χ^2^ (1) = 0.96, *p* = 0.328	26.3%	64.6%	χ^2^ (1) = 24.04, *p* < 0.001
Who sent it to you? (% different gender)	67.0%	68.4%	65.4%	χ^2^ (1) = 0.35, *p* = 0.552	60.6%	73.6%	χ^2^ (1) = 6.67, *p* = 0.010
Who did you send it to? (% different gender)	31.6%	25.8%	35.4%	χ^2^ (1) = 1.61, *p* = 0.205	31.2%	32.1%	χ^2^ (1) = 0.02, *p* = 0.900
Mean number of times disseminated a sext *(SD)*	3.69 (4.74)	3.92 (4.93)	3.54 (4.62)	*t* (155) = 0.50, *p* = 0.619	3.58 (4.34)	3.80 (5.12)	*t* (155) = −0.28, *p* = 0.777
Number of people to whom disseminated sext was sent	2.28 (4.56)	1.66 (1.21)	2.72 (5.83)	*t* (154) = −1.43, *p* = 0.154	2.21 (2.69)	2.36 (5.98)	*t* (154) = −0.21, *p* = 0.838
Ever had own sext disseminated	12.3%	13.4%	11.3%	χ^2^ (1) = 1.13, *p* = 0.287	9.4%	14.8%	χ^2^ (1) = 7.62, *p* = 0.006
Consent for image to be disseminated	15.2%	7.2%	23.2%	χ^2^ (1) = 6.80, *p* = 0.009	29.4%	6.9%	χ^2^ (1) = 12.63, *p* < 0.001
Unconditional respect for persons	5.43 (1.03)	5.55 (0.95)	5.36 (1.07)	*t* (914) = 2.58, *p* = 0.010	5.21 (1.02)	5.68 (.99)	*t* (914) = −7.00, *p* < 0.001
Subjective norms and attitudes to dissemination	8.74 (3.17)	8.58 (2.74)	8.87 (3.48)	*t* (975) = −1.45, *p* = 0.147	9.54 (3.41)	8.02 (2.75)	*t* (975) = 7.67, *p* < 0.001

Note: All percentages refer to participants answering in the affirmative.

**Table 2:** Sexting behaviours by gender within country.

Variable	Aus			U.S.		
	Men (*n* =233)	Women (*n* = 297)	Comparison, Aus Men to Women	Men (*n* = 307)	Women (*n* =311)	Comparison, U.S. Men to Women
Received sext	76.7%	90.6%	χ^2^ (1) = 18.68, *p* < 0.001	86.8%	89.0%	χ^2^ (1) = 0.67, *p* = 0.413
Received unwanted/unwelcome sext (% of above)	24.6%	77.4%	χ^2^ (1) = 120.82, *p* < 0.001	32.5%	74.3%	χ^2^ (1) = 95.14, *p* < 0.001
Requested a sext	53.8%	37.7%	χ^2^ (1) = 13.35, *p* < 0.001	62.7%	47.9%	χ^2^ (1) = 13.55, *p* < 0.001
Sent sext (of yourself)	51.6%	74.1%	χ^2^ (1) = 28.05, *p* < 0.001	61.6%	69.9%	χ^2^ (1) = 4.66, *p* = 0.031
Received disseminated sext	38.6%	33.7%	χ^2^ (1) = 1.40, *p* = 0.238	26.4%	24.1%	χ^2^ (1) = 0.42, *p* = 0.516
Last time received a disseminated sext: was receiving image unexpected or unwelcome	30.8%	63.0%	χ^2^ (1) = 19.84, *p* < 0.001	45.7%	72.4%	χ^2^ (1) = 11.51, *p* = 0.001
Ever disseminated sext	11.2%	14.1%	χ^2^ (1) = 1.04, *p* = 0.308	17.6%	12.9%	χ^2^ (1) = 2.68, *p* = 0.102
Last time disseminated a sext, was receiving image unexpected or unwelcome (% of above)	7.7%	61.9%	χ^2^ (1) = 19.49, *p* < 0.001	35.2%	67.5%	χ^2^ (1) = 9.60, *p* = 0.002
Mean number of times disseminated a sext (SD)	5.00 (6.74)	3.32 (3.50)	t (62) = 1.32, *p* = 0.192	2.98 (2.62)	4.31 (6.41)	t (91) = −1.37, *p* = 0.173
Mean number of people to whom sext was sent (SD)	1.81 (1.27)	1.55 (1.18)	t (62) = 0.83, *p* = 0.412	2.40 (3.14)	3.19 (8.41)	t (90) = −0.63, *p* = 0.527
Ever had own sext disseminated	10.3%	15.8%	χ^2^ (1) = 3.44, *p* = 0.064	8.8%	13.8%	χ^2^ (1) = 3.89, *p* = 0.048
Consent for image to be disseminated	12.5%	4.4%	χ^2^ (1) = 1.51, *p* = 0.219	44.4%	9.5%	χ^2^ (1) = 11.25, *p* = 0.001
Unconditional respect for persons (URfP)	5.38 (0.95)	5.76 (0.90)	t (334) = −3.64, *p* < 0.001	5.10 (1.05)	5.63 (1.03)	t (578) = −6.20, *p* < 0.001
Subjective norms and attitudes to dissemination (SNA)	9.31 (3.03)	7.95 (2.29)	t (334) = −3.64, *p* < 0.001	9.71 (3.68)	8.09 (3.0)	t (530) = 5.53, *p* < 0.001

Note: All percentages refer to participants answering in the affirmative.

**Table 3:** Correlations for key variables: upper triangle represents Australian participants, lower triangle represents U.S. participants.

Variables	1	2	3	4	5	6	7	8	9	10	11	12	13	14
Dissemination	-	0.04	0.04	0.15 **	0.17 **	0.18 **	0.13 **	a	0.17 **	−0.15 *	0.30 **	0.01	0.24 **	−0.07
Age	0.06	-	−0.14 **	0.02	−0.15 **	0.17 **	−0.01	0.01	−0.03	−0.30 **	−0.04	0.14	0.07	0.17 **
Gender	−0.07	0.00	-	0.19 **	0.52 **	−0.16 **	0.23 **	0.54 **	−0.05	0.32 **	0.08	−0.15	−0.25 **	0.20 **
Received sext	0.09 *	0.08	0.03	-	b	0.32 **	0.56 **	0.10	0.21 **	−0.01	0.15 **	0.03	−0.07	0.09
Received unwanted	0.16 **	−0.06	0.42 **	b	-	−0.20 **	0.04	0.40 **	0.03	0.29 **	0.13 **	−0.07	−0.18 **	0.09
Requested sext	0.18 **	−0.02	−0.15 **	0.36 **	−0.11 *	-	0.41 **	−0.32 **	0.13 **	−0.12	0.18 **	0.18	0.04	−0.02
Sent sext	0.14 **	−0.06	0.09 *	0.47 **	−0.02	0.56 **	-	−0.04	0.16 **	−0.08	0.232 **	0.08	−0.07	0.13 *
Dissemination: Receipt of image unwanted	a	0.06	0.32 **	−0.24 *	0.44 **	−0.20	−0.17	-	−0.31 *	0.16	0.18	−0.31	−0.39 **	0.23
Dissemination Receipt	0.19 **	0.01	−0.03	0.08	0.14 **	0.14 **	0.09 *	0.15	-	−0.08	0.18 **	0.12	0.12 *	−0.05
Dissemination Receipt: image unwanted	−0.11	−0.05	0.27 **	−0.06	0.26 **	−0.12	−0.01	0.58 **	−0.07	-	0.04	0.00	−0.33 **	0.13
Dissemination Victimisation	0.22 **	0.01	0.08 *	0.13 **	0.23 **	0.17 **	0.22 **	0.11	0.25 **	0.03	-	c	0.03	−0.08
Dissemination victimisation: Consensual	0.37 **	0.20	−0.40 **	a	0.07	0.03	−0.16	0.30	0.15	0.04	c	-	0.00	0.02
Dissemination SNA	0.29 **	0.00	−0.23 **	0.12 **	−0.02	0.09 *	−0.03	0.03	0.11 *	−0.08	0.14 **	0.42 **	-	−0.41 **
Unconditional Respect for persons	−0.11 **	−0.02	0.25 **	−0.04	0.05	−0.01	0.12 **	−0.12	−0.02	0.02	−0.01	−0.45 **	−0.47 **	-

a. Not calculated, as receiving unwanted disseminated images is a subset of dissemination; b. not calculated, as receiving unwanted images is a subset of receiving images; c. not calculated as consensual dissemination victimisation is subset of dissemination victimisation; * *p* < 0.05; ** *p* < 0.01.

**Table 4:** Motivations for sext dissemination by country and gender.

Motives	OVERALL (*N* = 162)				Australian (*N* = 68)				U.S. (*N* = 94)			
	Total	Men (*N* = 80)	Women (*N* = 82)	Comparison	Overall	Men (*N*= 26)	Women (*N*= 42)	Comparison	Overall	Men (*N*= 54)	Women (*N* = 40)	Comparison
Because the person in the image was hot	70 (42.9%)	52 (64.2%)	18 (22.0%)	χ^2^ (1) = 29.68, *p* < 0.001	30 (43.5%)	20 (74.1%)	10 (23.8%)	χ^2^ (1) = 16.90, *p* < 0.001	40 (42.6%)	32 (59.3%)	8 (20.0%)	χ^2^ (1) = 14.49, *p* < 0.001
As a joke, to be funny	46 (28.4%)	20 (25.0%)	26 (31.7%)	χ^2^ (1) = 0.90, *p* = 0.344	23 (33.8%)	8 (30.8%)	15 (35.7%)	χ^2^ (1) = 0.18, *p* = 0.794	23 (24.5)	12 (22.2%)	11 (27.55)	χ^2^ (1) = 0.35, *p* = 0.556
To gossip	37 (22.8%)	11 (13.8%)	26 (31.7%)	χ^2^ (1) = 7.41, *p* = 0.006	20 (29.4%)	5 (19.2%)	15 (35.7%)	χ^2^ (1) = 2.10, *p* = 0.147	17 (18.1%)	6 (11.1%)	11 (27.5%)	χ^2^ (1) = 4.17, *p* = 0.041
I did not think it was a big deal	32 (19.8%)	14 (17.5%)	18 (22.0%)	χ^2^ (1) = 0.51, *p* = 0.477	15 (22.1%)	6 (23.1%)	9 (21.4%)	χ^2^ (1) = 0.03, *p* = 0.873	17 (18.1%)	8 (14.8%)	9 (22.5%)	χ^2^ (1) = 0.92, *p* = 0.338
To brag	24 (14.8%)	18 (22.5%)	6 (7.3%)	χ^2^ (1) = 7.40, *p* = 0.007	13 (19.1%)	10 (38.5%)	3 (7.1%)	*	11 (11.7%)	8 (14.8%)	3 (7.5%)	*
To roast or tease the person depicted	22 (13.6%)	2 (2.5%)	20 (24.4%)	*	7 (10.3%)	2 (7.7%)	5 (11.9%)	*	15 (16.0%)	0	15 (37.5%)	*
To improve your social status	15 (9.3%)	13 (16.3%)	2 (2.4%)	χ^2^ (1) = 9.19, *p* = 0.002	6 (8.8%)	4 (15.4%)	2 (4.8%)	*	9 (9.6%)	9 (16.7%)	0	*
Because another person asked you to	13 (8.0%)	10 (12.5%)	3 (3.7%)	χ^2^ (1) = 4.29, *p* = 0.038	5 (7.45)	4 (15.4%)	1 (2.4%)	*	8 (8.5%)	6 (11.1%)	2 (5.0%)	*
Out of spite	12 (7.4%	5 (6.3%)	7 (8.5%)	χ^2^ (1) = 0.31, *p* = 0.578	6 (8.8%)	2 (7.7%)	4 (9.5%)	*	6 (6.4%)	3 (5.6%)	3 (7.5%)	*
To initiate sexual contact	11 (6.8%)	5 (6.3%)	6 (7.3%)	χ^2^ (1) = 0.07, *p* = 0.787	3 (4.4%)	1 (3.8%)	2 (4.8%)	*	8 (8.5%)	4 (7.4%)	4 (10.0%)	*
To get attention/praise	10 (6.2%)	6 (7.5%)	4 (4.9%)	*	7 (10.3%)	3 (11.5%)	4 (9.5%)	*	3 (3.2%)	3 (5.6%)	0	*
To get back at the person/get revenge	7 (4.3%)	0	7 (8.5%)	*	2 (2.9%)	0	2 (4.8%)	*	5 (5.3%)	0	5 (12.5)	*
To get recipient into trouble	5 (3.1%)	3 (3.8%)	2 (2.4%)	*	0	0	0	*	5 (5.3%)	3 (5.6%)	2 (5.0%)	*
Because you felt pressured to do so	2 (1.2%)	2 (2.5%)	0	*	1 (1.5%)	1 (3.8%)	0	*	1 (1.1%)	1 (1.9%)	0	*

* Chi-square analyses not conducted where cell counts < 5 as expected cell counts too low.

**Table 5:** Regression results for overall sample.

Independent Variables	B	*p*	Exp(B)	95% CI’s	
				Lower	Upper
Gender	0.29	0.71	1.34	0.28	6.35
Age	0.06	0.23	1.07	0.96	1.19
Country *	−3.19	0.02	0.04	0.00	0.66
Received sext	0.30	0.61	1.35	0.42	4.31
Requested sext	0.59	0.04	1.80	1.02	3.18
Sent sext	0.36	0.29	1.44	0.73	2.83
Received disseminated sext	0.74	0.003	2.09	1.28	3.41
Own sexts disseminated	1.34	<0.001	3.83	2.19	6.68
Subjective norms and attitudes to dissemination	0.28	0.01	1.32	1.06	1.64
Respect for persons	0.13	0.33	1.14	0.88	1.48
Gender * Country	1.63	0.003	5.11	1.72	15.21
Gender * Subjective norms and attitudes	−0.04	0.56	0.96	0.83	1.10
Country * Subjective norms and attitudes	0.08	0.30	1.09	0.93	1.27
Constant	−8.13				

* Reference category country = U.S.

## Data Availability

Aggregated data supporting these results can be provided by the authors on request.

## Supplementary Materials

The following are available online at https://www.mdpi.com/1660-4601/18/5/2429/s1, Sexting Behaviours Questionnaire.

## Appendix A

Seven items were initially included for about subjective norms and attitudes towards sext dissemination, as indicated. Two items (Items 3 and 4) were poorly correlated with the overall scale, and were removed from the full scale.

**Table A1:** Item and scale totals and correlations for Subjective Norms and Attitudes to Sext Dissemination Scale (SNA).

	Item Wording	Mean	SD	Scale Mean if Item Deleted	Scale Variance if Item Deleted	Corrected Item-Total Correlation	Squared Multiple Correlation	Cronbach’s Alpha if Item Deleted
1	Forwarding sexually explicit images via text or mobile app is no big deal.	1.90	1.07	11.43	10.96	0.35	0.19	0.44
2 *	Forwarding sexually explicit images via text or mobile app can have serious negative consequences.	1.62	0.93	11.72	11.16	0.41	0.21	0.42
3 *	Sexually explicit images via text or mobile app usually end up being seen by more than just those to whom they were sent.	2.44	1.22	10.90	12.12	0.11	0.07	0.55
4 *	Females have to worry more than males about sexually explicit images of themselves being viewed or distributed via text or mobile app to someone other than they were intended for.	2.15	1.32	11.19	11.85	0.10	0.08	0.56
5	Sharing sexually explicit images via text or mobile app of others can enhance social status.	2.26	1.13	11.08	11.76	0.20	0.14	0.50
6	After a relationship breakdown, it is acceptable to forward sexually explicit images of your ex via text or mobile app to others.	1.29	0.76	12.04	11.84	0.41	0.29	0.44
7	Forwarding or sharing sexually explicit images of others via text or mobile app can be funny.	1.68	1.00	11.66	11.16	0.36	0.32	0.44

* Item reverse coded prior to scale testing and construction.
