# Supplementary material for: Sext Dissemination: Differences across Nations in Motivations and Associations
Source: Int J Environ Res Public Health. 2021 Mar 2;18(5):2429. doi: 10.3390/ijerph18052429 (PMC7967565; doi:10.3390/ijerph18052429)
Supplement: Supplementary file 1 [file ijerph-18-02429-s001.pdf]

Sext dissemination Behaviours Questionnaire

Initial statement: For the purposes of this survey, sexts are defined as sexually explicit image via text message or mobile app.

RECEIVING

1. **Have you ever received an image-based sext?**
   Yes /No
   If yes, how many times have you received an image-based sext?
   1-2 times
   3-5 times
   6-10 times
   11 to 25 times
   More than 25 times

RECEIVING – Unsolicited/Unwanted and impacts

2. **Have you ever received an image-based sext that was unsolicited or unexpected?**
   Yes /No

3. **Have you ever received an image-based sext that was unwanted/unwelcome?**
   Yes /No

4. **If yes, approximately how many times has this happened in the past 12 months (approx.)**
   a. **How do you mostly feel after having received the unwanted or unwelcome image-based sext?** (You may select as many items as applicable)

      Aroused Curious Angry Embarrassed Afraid Flattered
      Ashamed Neutral Violated Amused Other (____)

   a. **How distressed do you usually feel about receiving an unwanted or unwelcome image-based sext?**

0	1	2	3	4	5
Not at all	Very slightly	Slightly	Moderately	Strongly	Very strongly

1. **Thinking about the last time you received an image-based sext, was receiving that picture unwanted or unwelcome?**

   Yes/No

   a. **From whom did you receive the image-based sext?**

Friend (same sex)	Friend (opposite sex)	Boyfriend/ girlfriend	Ex-boyfriend/ girlfriend	Someone you just met	Someone you only knew online	Stranger

b. **Was the person in the image of a gender you are attracted to?**

c. **How did you mainly feel after having received an image-based sext?** (You may select as many items as applicable)

Aroused Curious Angry Embarrassed Afraid Flattered
Ashamed Neutral Violated Amused Other (____)

d. **How distressed were you?**

0	1	2	3	4	5
Not at all	Very slightly	Slightly	Moderately	Strongly	Very strongly

e. **Did you feel pressure to respond?**

Yes/No

f. **Did you reciprocate by sending an image-based sext to the sender?**

Yes/No

g. **Were you worried that this might lead to future harassment from this person?**

Yes/No

REQUESTING

2. **Have you ever asked someone to send you a sexually explicit image of themselves via text or mobile app?**

Yes / No

a. **Have you ever hassled someone to send you a sexually explicit image of themselves via text or mobile app (e.g. asked repeatedly or was pushy)?**

Yes / No

b. **What was the outcome?**

No response
Was told off
Abuse
Received a sext

Other (________________)

DISSEMINATION - Perpetration

3. **Have you ever received a sexually explicit image sent via text or mobile app intended for yourself which you subsequently distributed to another person (this includes showing or sharing the image)?**
   Yes /No
   a. **If yes, how many times have you distributed? (If you can't remember, try to estimate as best as you can…….) ________________**__________(Numerical response only)

4. **Think about the last time you distributed an image you had received….(not yourself)**
   a) **Who sent it to you (female/male/other)?**

   b) **Was the person who sent it to you, also the person depicted in the image?** (Yes/No)

      a. **If yes, were you romantically involved with that person when you <u>received</u> the image?**

         Yes/no

      b. **Were you romantically involved with that person when you distributed the image?**

         Yes/no

      c. **Was it unexpected/unwelcome?**
         Yes/No

   c) **Approximately how many people did you send the image to?**

      Numerical response ______________

   d) **Did you send it to….?**

      Males / Females / Males and Females

   e) **What were the reasons why you decided to share the sext message with others? (You may select more than one answer).**

As a joke, to be funny

Because the person in the image was hot

To get attention/praise

Out of spite

To gossip

Because another person asked you to	Because you felt pressured to do so
To improve your social status amongst peers	To get the recipient into trouble
To initiate sexual contact	To get back at the person / to get revenge
To roast or tease the person depicted	To brag
I did not think it was a big deal	Other (___________)

f) **Did you think there might be negative consequences for the person depicted in the image?**
Yes/No/Not sure

g) **What was the response you received to distributing this image?**

- Receiving images in return
- Sexual contact
- Praise
- Abuse
- Told me off
- No response

DISSEMINATION – Receipt of disseminated images

5. **Has someone ever forwarded you a sexually explicit image via text or mobile app that was not originally intended for you?**

   Yes/No

   a. **Was it unwanted/unwelcome?**
   Yes/No

   b. **Who sent it to you?**

   Female/male/other

DISSEMINATION – Victimisation

6. **Have you ever sent a sexually explicit image of yourself via text message or mobile app that was subsequently forwarded (to your knowledge)?**

   Yes / No

   a. **Had you given permission for this image to be forwarded?**

   Yes / No

   b. **How distressed were you?**

0	1	2	3	4	5
Not at all	Very slightly	Slightly	Moderately	Strongly	Very strongly

SENDING

7. **Have you ever sent sexually explicit images of yourself via text message or mobile app?**

   Yes / No

   a. **If yes, how many times have you sent sexually explicit images of yourself via text message or mobile app?**

      1-2 times
      3-5 times
      6-10 times
      11 to 25 times
      More than 25 times

   b. **If yes, whom did you send the sexually explicit images of yourself to?** (You may select as many items as applicable)

Friend (same sex)	Friend (opposite sex)	Boyfriend/ girlfriend	Ex-Boyfriend/ girlfriend	Someone you just met	Someone you only knew online	Stranger

   c. **If you have sent sexually explicit images of yourself via text or mobile app to another person what was the reason/s?** (You may select as many items as applicable)

As a form of self-expression	As a joke, to be funny	To get attention/praise
To be flirtatious/fun	To be sexy/initiate sexual activity	Because another person asked you to
Because you felt pressured to do so	To improve your social status amongst peers	Bullying/harassment
To get the recipient into trouble	Other reason_______________	Don't know

8. **Have you ever consented to sexting (sent a sexually explicit image via text or mobile app) when you actually did not want to sext?**

Yes /No

a. **If yes, please indicate whether the following occurred:**

	Strongly agree (1)	(2)	(3)	(4)	(5)	Strongly disagree (6)
**(i). The recipient pressured me to sext them**						
**(ii). They persisted in asking me to sext them, even though they knew I did not want to**						
**(iii). They made me feel obligated to sext them**						
**(iv). The recipient *told me* that if I were truly committed, I would sext**						
**(v). The recipient *told me* that if I loved him/her I would sext**						
**(vi). The recipient *told me* that it was my obligation or duty to sext him/her**						
**(vii). The recipient *told me* he/she would sext another person if I did not sext him/her**						
**(viii). The recipient threatened to pursue a relationship with someone else if I did not sext him/her**						
**(xi). The recipient told me that others were interested in sexting him/her, so that I would sext**						

b. **How distressed were you?**

0	1	2	3	4	5
Not at all	Very slightly	Slightly	Moderately	Strongly	Very strongly

9. **Are you aware of any negative consequences that may arise as a result of sending/receiving sexually explicit images via text message or mobile app?** (You may select as many items as applicable)

Bullying/Harassment	Blackmail	Legal consequences	Affecting job prospects
Affecting current romantic relationship	Affected relationship with parents	Showing/sending of the image without your consent	Other (please specify)

10. **Have you ever considered sending a sexually explicit image of yourself to another person via text message or mobile app and decided against it?**

Yes /No

a. **If yes, what was your reason for deciding against it?**
   Fear of legal consequences
   Getting into trouble at school
   Fear of parents finding out
   Not liking my body enough
   Fear of response of others
   Fear of it being sent to a third party
   Not liking being pressured into it
   Other ______________
   Don’t want to say

11. **Have you, or someone else you know, experienced negative consequences as a result of sending sexually explicit images of themselves to another person via text message or mobile app?**

    Yes /No

    a. **If yes, who experienced these negative consequences?**
       Self
       Other person
    b. **If yes, what negative consequences were experienced?** (You may select as many items as applicable)

Bullying/Harassment	Blackmail	Legal consequences	Affecting job prospects
Affecting current relationship	Affected relationship with parents	Other - ________________	Showing/sending of the image without your consent

12. **On average, how likely do you think it is that somebody would distribute/forward on a sexually explicit image that you sent them of yourself?**
